# Patient-Specific Analysis of Ascending Thoracic Aortic Aneurysm with the Living Heart Human Model

**DOI:** 10.3390/bioengineering8110175

**Published:** 2021-11-04

**Authors:** Salvatore Cutugno, Valentina Agnese, Giovanni Gentile, Giuseppe M. Raffa, Andrew D. Wisneski, Julius M. Guccione, Michele Pilato, Salvatore Pasta

**Affiliations:** 1 Department of Engineering (DING), Università Degli Studi di Palermo, Viale Delle Scienze Ed.8, 90128 Palermo, Italy; salvatore.cutugno@unipa.it; 2 Department for the Treatment and Study of Cardiothoracic Diseases and Cardiothoracic Transplantation, IRCCS-ISMETT, 90127 Palermo, Italy; vagnese@ismett.edu (V.A.); graffa@ismett.edu (G.M.R.); mpilato@ismett.edu (M.P.); 3 Department of Diagnostic and Therapeutic Services, IRCCS-ISMETT, 90127 Palermo, Italy; gigentile@ismett.edu; 4 Department of Surgery, University of California San Francisco, San Francisco, CA 94143, USA; andrew.wisneski@ucsf.edu (A.D.W.); Julius.Guccione@ucsf.edu (J.M.G.)

**Keywords:** ascending aortic aneurysm, cardiac mechanics, finite element analysis, living heart human model

## Abstract

In ascending thoracic aortic aneurysms (ATAAs), aneurysm kinematics are driven by ventricular traction occurring every heartbeat, increasing the stress level of dilated aortic wall. Aortic elongation due to heart motion and aortic length are emerging as potential indicators of adverse events in ATAAs; however, simulation of ATAA that takes into account the cardiac mechanics is technically challenging. The objective of this study was to adapt the realistic Living Heart Human Model (LHHM) to the anatomy and physiology of a patient with ATAA to assess the role of cardiac motion on aortic wall stress distribution. Patient-specific segmentation and material parameter estimation were done using preoperative computed tomography angiography (CTA) and ex vivo biaxial testing of the harvested tissue collected during surgery. The lumped-parameter model of systemic circulation implemented in the LHHM was refined using clinical and echocardiographic data. The results showed that the longitudinal stress was highest in the major curvature of the aneurysm, with specific aortic quadrants having stress levels change from tensile to compressive in a transmural direction. This study revealed the key role of heart motion that stretches the aortic root and increases ATAA wall tension. The ATAA LHHM is a realistic cardiovascular platform where patient-specific information can be easily integrated to assess the aneurysm biomechanics and potentially support the clinical management of patients with ATAAs.

## 1. Introduction

An ascending thoracic aortic aneurysm (ATAA) is diagnosed in approximately 10 out of 100,000 persons per year in developed countries [1]. The condition is particularly lethal with an estimated 5 year mortality rate of 39% for aortic diameters ≤6 cm and 62% for ATAAs ≤6 cm [2]. Elective surgical repair of ATAA is a major operation that carries high morbidity and mortality; however, if the dilated ascending aorta is left untreated, spontaneous aortic rupture or dissection may occur. Current clinical guidelines suggest that the threshold governing the timing for optimal intervention of surgical repair is a diameter of 5.5 cm [3]. Nevertheless, observational studies suggest that >50% of acute Type A dissections have an aneurysm size below the surgical threshold [4]. The prognostic capability of the maximum diameter criterion may be poor because it considers the high risk of spontaneous dissections in patients with genetic predisposition, population statistics, and inconsistent use of imaging methods for diagnosis [5].

Current research on ATAAs focuses on the development of novel predictors of aneurysm development and failure, as well as the understanding of the mechanistic link between wall weakening and vessel function [6,7]. New morphometric classification schemes based on aortic shape and valve phenotype [8], flow analysis based on cardiac MRI [9,10] and on computational modeling [11,12,13,14,15], and novel circulating biomarkers [16,17,18] have been proposed to support the clinical risk stratification in patients with ATAAs. Notwithstanding the efforts of many research groups on identifying novel metrics not based on aortic size, the optimal timing for aneurysm intervention remains a challenging question.

Computational modeling techniques including structural finite-element analysis [19,20], computational fluid dynamics [21,22], and fluid–solid interaction [23,24] have been widely adopted in the last decade to assess ATAA biomechanics. Wall stress predictions and their correlations with anatomical sites of rupture and dissection demonstrated the value of wall stress magnitude over aortic size measurement [25]. There were, however, technical challenges in modeling ATAAs such that simplification and assumptions were necessary to describe the mathematical models of aneurysm mechanics. For instance, ATAA wall thickness is not easily measured on clearly preoperative computed tomography (CT) scans and has often resulted in the aorta being modeled as a shell with uniform thickness. This approach does not allow the analysis on the risk of dissection from the separation of aortic wall layers. Moreover, blood flow was modeled as hydrostatic uniform, or FSI-derived pressure was the most common boundary condition loading the ATAA wall. However, the heartbeat leads to a stretch and twist of the ATAA wall that increases the intramural stress at a level that is likely more relevant than the aortic blood pressure. Indeed, CT scanning has demonstrated that the ATAA, often stiffer than non-aneurysmal aorta, exhibits a small change in the cross-sectional area between systole and diastole [26,27], while the aorta could be stretched upon 8.9 mm downward from the contracting myofiber at systole [28]. 

In this study, we sought to determine the biomechanics of ATAAs in the setting of a beating heart to assess the impact of aortic root motion and quantify the aortic wall stress along the longitudinal, circumferential, and transmural directions. To accomplish this, we used the realistic and accurate multiphysics Living Heart Human Model (LHHM) developed by Dassault Systèmes [29] to provide a new technological platform for aortic aneurysm modeling. We modified the original LHHM to include the patient-specific aneurysm geometry, material descriptors, and vascular physiology and compared the simulation results of the LHHM with and without patient-specific ATAA.

## 2. Materials and Methods

**Figure 1:**
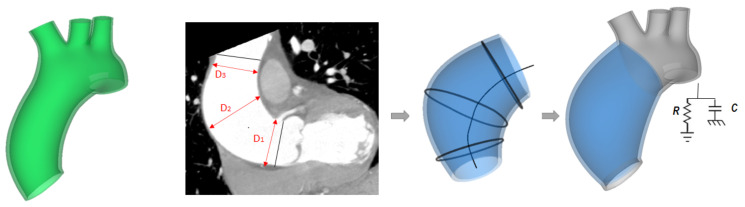
Workflow showing the modification of the original LHHM to account for the ATAA; the green model (**left**) shows the original aortic model; measurements of ATAA diameter from CT images (**middle-left**); ATAA model showing the centerline and curve used for the loft protrusion surface (**middle-right**); ATAA model showing the coupling with the lumped-parameter model as boundary condition (**right**).

**Figure 2:**
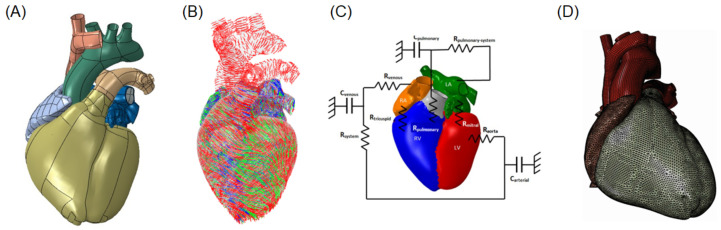
Sketch of LHHM implementation: (**A**) heart and great vessel geometries; (**B**) fiber architecture; (**C**) coupling with 1D lumped-parameter model; (**D**) structural mesh.

The proposed computational workflow to include the ATAA in the LHHM consisted of three steps: (1) integration of the patient-specific ascending aneurysm anatomy (see Figure 1), (2) assessment of constitutive material descriptors from biaxial testing, and (3) adaptation of the lumped-parameter model of systemic circulation using clinical data collected during patient workup (see Figure 1).

### 2.1. Patient Study Case 

The ATAA geometry was obtained from the preoperative computed tomography angiography (CTA) images of a 70 year old man with an ascending aortic diameter of 54 mm, scheduled to undergo elective surgical repair of his ATAA The resected ATAA tissue was stored at −80 °C and then subjected to biaxial stretch testing for the estimation of constitutive material descriptors. At in-hospital admission, demographic data and cuff pressure measurement were collected. The heart function, such as the stroke volume and cardiac output, was assessed by echocardiography. Patient parameters were a systolic pressure of 132 mmHg, diastolic pressure of 84 mmHg, mean arterial pressure of 102 mmHg, stroke volume of 64 mL, cardiac output of 4.9 L/min, and heart rate of 60 bpm. A tri-leaflet aortic valve with normal function (orifice area of 414 mm^2^) was found. The study was approved by our local research ethics committee, and the patient signed the informed consent form before surgery.

### 2.2. ATAA Reconstruction

CTA images reconstructed at end diastole were analyzed using the segmentation software Mimics Innovation Suite (v.22, Materialise, Leuven, Belgium). Specifically, the mask of the aortic wall was semiautomatically reconstructed using thresholding, region growing, and then manual editing as described previously [30,31]. The aortic centerline was marked, followed by the contour of the inner aortic lumen by spline curves generated at several anatomic levels (see Figure 1). The inner aortic luminal surface was determined by loft protrusion of the aortic contour lines using the centerline as a rail curve. The aortic wall was obtained as offset of the inner aortic surface in Rhinoceros software (McNeel & Associates, Seattle, WA, USA), assuming a thickness of 2.1 mm. The ATAA model was then meshed in ICEM software (Ansys v.21, ANSYS Inc., Canonsburg, PA, USA) using 199,960 unstructured tetrahedral solid elements (S3). Starting from the original LHHM geometry, the ascending aorta from the sino-tubular junction to the aortic arch was replaced with the ATAA mesh, thus leaving the aortic root and valve unmodified.

### 2.3. Biaxial Testing 

To adapt the biomechanical response of LHHM model, equibiaxial material testing was carried out on a square specimen (15 × 15 mm) cut from the harvested ATAA specimen as done previously by our group [32]. In brief, the specimen was anchored by sutures to the four electromagnetic motors of the ElectroForce TestBench system (TA Instrument, Boston, MA, USA), aligning circumferential and longitudinal edges with direction of stretching. Engineering strains were measured by a digital video extensometer monitoring the displacement of five black markers placed on the intimal tissue surface of the specimen. During material testing, the specimen was submerged in a bath with 0.9% isotonic saline solution under a controlled temperature of 37 °C. A small preload was set, and then preconditioning of the tissue sample at 7% strain was carried out. The test was characterized by a crosshead displacement speed of 1 mm/min and by two 200 N loads cells for recording the forces along stretching directions. Data were analyzed to achieve the Green strains and first Piola–Kirckhhoff in both circumferential and longitudinal directions. Fitting was performed using a custom nonlinear least-squares algorithm with trust-region-reflective developed in Matlab (v2021, Mathworks, Natick, MA, USA).

### 2.4. LHHM Model and Adaption to ATAA

The LHHM developed by the SIMULIA Living Heart Project (see Figure 2A) is a realistic and high-fidelity model of an adult male heart, featuring the anatomy of the four chambers, heart valves, and major vessels (i.e., the aorta, pulmonary artery, and vena cava) [33]. In this study, we modified the LHHM by replacing the original aortic model with the patient-specific ATAA geometry and then adapted the boundary conditions and aortic wall material properties (i.e., the pressure and flow of ATAA model) to include patient-specific information. The biomechanical response of LHHM is governed by an electrical activation of its Purkinje system, which in turn activates the myocardium. The myocardial material parameters include myocardial activation fiber architecture, as well as active and passive myocardial elements. The structural model is coupled to a 1D lumped-parameter model to account for the human blood circulation and its interaction with deforming heart chambers. All anatomical parts are meshed using 3D tetrahedral elements (Figure 2D) except chordae tendineae discretized with truss elements. The LHHM simulates the normal cardiac beat of an adult (60 bpm).

The electrical response is characterized by an action potential and recovery described by Hurtado and Kuhl [34] and was not modified in this study. While the cardiac fiber orientation follows a rule-based approach proposed by Dabiri et al. [35] to model fiber changes from endocardium to epicardium (Figure 2B), we changed the fiber orientation of the aneurysmal aorta (as compared to the original LHHM) using fiber angle distribution extrapolated by our previous investigation using multiphoton imaging of ATAA microarchitecture [36]. Specifically, fibers were oriented with an angle of 34° with respect to the circumferential direction as compared to the 42° fiber angle of the original LHHM. Both active contraction and passive material response are simulated in the LHHM using constitutive modeling. The active stress in the cardiac fiber direction is generated by a time-varying elastance model, where the active force is a function of the current sarcomere length, peak intracellular calcium concentration, and fiber activation time [37]. In a different way, the passive behavior of heart chambers is modeled with the anisotropic hyperelastic constitutive model proposed by Ogden and Holzapfel [38], which has been widely used in many cardiac simulation studies [37,39]. The strain energy function is represented by the following invariant-based formulation:

(1) $$\Psi_{dev}=\frac{a}{2b}\exp\left[b(I_{1}-3)\right]+\underset{i=f,s}{\sum }\frac{a_{i}}{2b_{i}}\left\{\exp\left[b_{i}({\left(I_{4i}-1\right)}^{2})\right]-1\right\}+\frac{a_{fs}}{2b_{fs}}\left[\exp,\left(b_{fs}I_{8fs}^{2}-1\right)\right]$$

Eight material parameters *a, b, a_f_, b_f,_ a_s_, b_s_, a_fs_*, and *b_fs_*, and four strain invariants *I_1_, I_4f_, I_4s_*, and *I_8fs_* define Equation (1) for heart chambers and great vessels. In this study, only the passive anisotropic behavior was considered for the ATAA, with the *a_fs_* and *b_fs_* material parameters set to null. Thus, the material parameters in our LHHM-modified version including the ATAA model were replaced with those derived by the fitting of biaxial stress-strain curves of the investigated patient case. To account for viscoelastic properties of human tissues, the LHHM includes an isotropic time-dependent linear viscoelastic response as part of the constitutive behavior of the heart.

The blood flow inside the heart chambers and great vessels integrates a hybrid approach involving 3D representations based on the fluid-cavity approach and lumped-parameter modeling of the pulmonary and systemic circulatory systems (see Figure 2C). We modified the initial values of the compliance and resistance of the LHHM arterial system using patient physiological data. Specifically, the total resistance of the arterial system was calculated as R=MAP/Q, where MAP is the patient mean arterial pressure and Q is the cardiac output as determined from blood pressure cuff measurement and echocardiography, respectively. The arterial compliance was C=ΔV/ΔP, where ΔV is the stroke volume indexed by the body surface area and ΔP is the blood pressure difference between systole and diastole. This numerical strategy allowed us to change the aortic pressure waveform of LHHM to match the systolic and diastolic cuff pressure measurement of the patient with ATAA. The cardiac output of the modified LHHM model was close to that of the original LHHM and was not modified.

The cardiac cycle of LHHM is simulated by a dynamic analysis in the ABAQUS/Explicit software (ABAQUS v2020, SIMULIA Inc., Providence, RI, USA). The left ejection fraction is set to 60% with an end-diastolic volume of 136–138 mL. This functional parameter of the left ventricle was not modified to represent a non-remodeled heart condition. A complete cardiac cycle occurred over 0.8 s, corresponding to a heart rate of 75 beats per minute. The systolic phase was 0.3 s long, while the simulation was started at 70% of ventricular diastole. The LHHM is constrained in space by fixed node sets at cut planes of the ATAA, pulmonary trunk, and superior vena cava. Therefore, the distal ends of supra-aortic vessels and descending aorta of the ATAA are allowed to move relative to fixed reference points such that the maximum motion of the cut planes is less than a few millimeters during the cardiac cycle. The approach is based on two parameters (Kelvin–Voigt model with a damper and an elastic spring) that are tuned to mimic the response of the physiological vessel motion. The values of these parameters are calculated from dynamic cardiac CT as described by Baillargeon et al. [32]. This allowed us to consider the compliance of the distal vasculature and obtain a physiological aortic motion. An acceptable steady-state solution was achieved after running three consecutive cardiac cycles to reduce the viscoelastic effects on the resulting stress distribution. The solution was considered stable when the stress changes was ≤10%. Structural and hemodynamic parameters of ATAA wall of the so-adapted LHHM were obtained over the cardiac cycle.

## 3. Results

**Figure 3:**
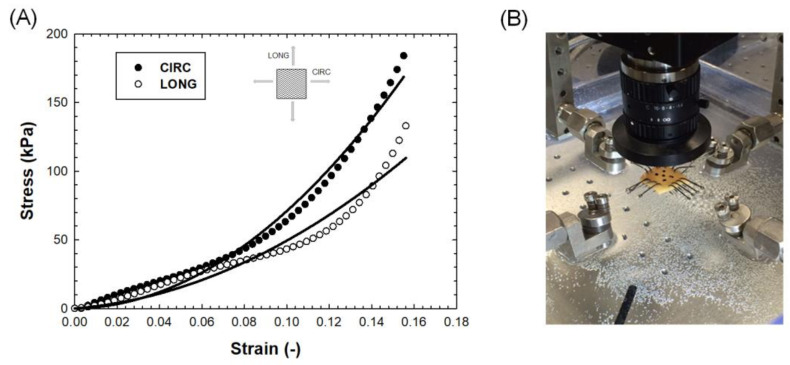
(**A**) Experimental stress–strain data (dots) and fitting (solid lines) from biaxial testing; (**B**) photography of experimental setup.

Figure 3 shows the experimental test and resulting raw data of the equibiaxial biomechanical response of the ATAA tissue specimen and the curve fitting using the Ogden and Holzapfel formulation. Stress–strain data had a nonlinear and hyperelastic mechanical response, which is dependent on the stretching direction (i.e., anisotropy). The constitutive model fit the experimental data well, as evinced by a correlation coefficient of 0.93 and normalized root-mean-square error of 0.375. Material descriptors can be considered effective upon an engineering strain of 0.18, and this span is likely caused by the premature failure of the tissue specimen at hooks. Material descriptors were $a=0.24\;\mathrm{MPa},\;b=6.6,\;a_{f}=0.05\mathrm{MPa},\;b_{f}=5.0,\;a_{s}=0.02\mathrm{MPa},\;b_{s}=2.0$. These values were found to be higher than the material parameters adopted for the healthy aorta in the unmodified LHHM (e.g., 60% increase for *a* and 230% for *b*), suggesting increased stiffness of the ATAA tissue compared to normal aorta. The increased stiffness is likely due to the disease progression and biomechanical derangement induced by aging. The latter cannot be considered in the LHHM simulation platform.

**Figure 4:**
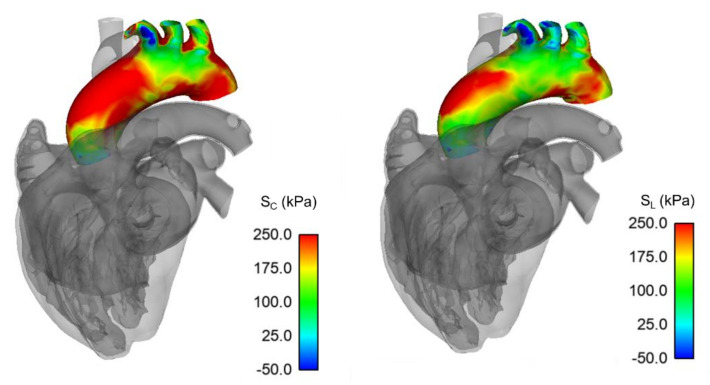
Circumferential (*S_C_*) and longitudinal stress (*S_L_*) map during systolic peak for unmodified LHHM healthy aorta.

**Figure 5:**
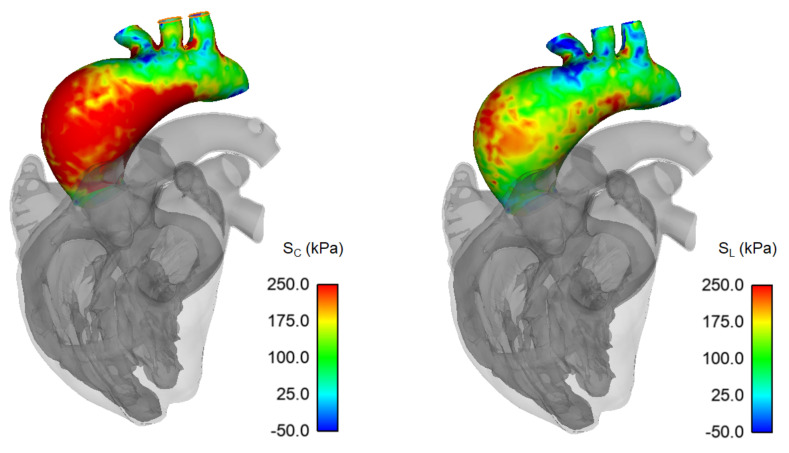
Circumferential (*S_C_*) and longitudinal stress (*S_L_*) map during systolic peak for ATAA case.

Figure 4 and Figure 5 show the systolic distribution of circumferential and longitudinal stresses at the inner aortic surface of both the normal aorta and ATAA LHHM simulations, respectively. The region where the circumferential stress peak exceeded 250 kPa was slightly more pronounced for the ATAA model than for the healthy aorta. In both cases, the circumferential stress was higher than stress in the longitudinal direction, with a homogeneous distribution around the vessel periphery. The greatest longitudinal stress was in the mid-ascending aorta along the major vessel curvature, as a result of the stretching and twisting induced by the cardiac cycle motion.

**Figure 6:**
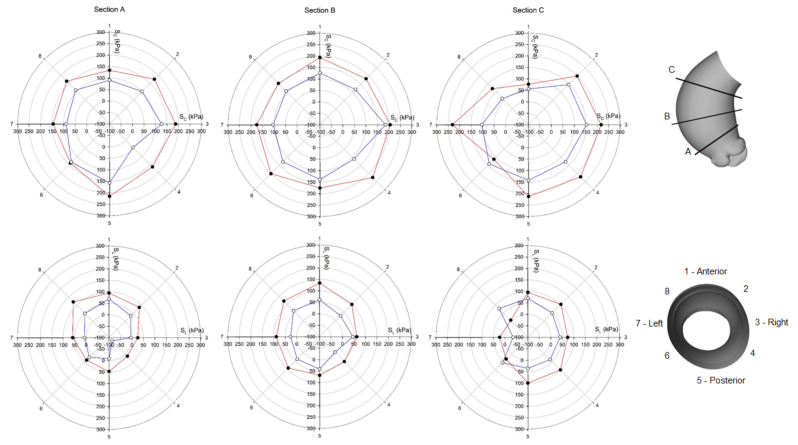
Polar plot of stress distribution along three different sections for ATAA LHHM at systolic peak for both the inner aortic wall surface (red lines) and the outer aortic wall surface (blue lines).

**Figure 7:**
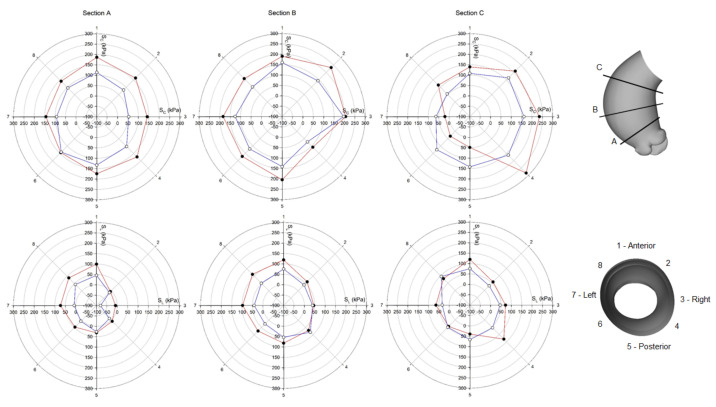
Polar plot of stress distribution along three different sections for ATAA LHHM at late diastole for both the inner aortic wall surface (red lines) and the outer aortic wall surface (blue lines).

**Figure 8:**
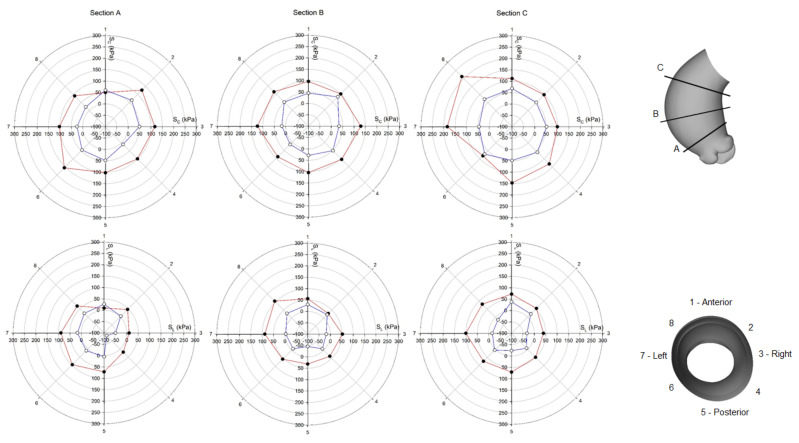
Polar plot of stress distribution along three different sections for healthy LHHM at systolic peak for both the inner aortic wall surface (red lines) and the outer aortic wall surface (blue lines).

**Figure 9:**
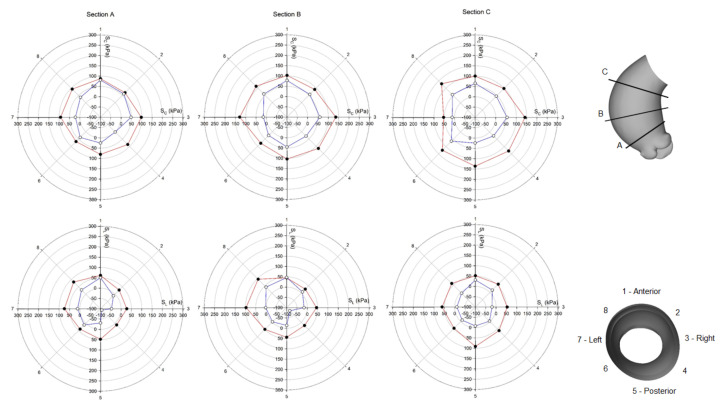
Polar plot of stress distribution along three different sections for healthy LHHM at late diastole for both the inner aortic wall surface (red lines) and the outer aortic wall surface (blue lines).

Using polar plots, Figure 6 and Figure 7 display the changes of circumferential and longitudinal stresses at both systole and diastole along the vessel circumference at three anatomic levels for the ATAA. Similarly, Figure 8 and Figure 9 show the polar plots for the healthy scenario. Section A represents a cross-section near the sino-tubular junction, section B was generated at the mid-ascending aorta, and section C represents just before the brachiocephalic trunk. Each section was divided into eight quadrants with the first one corresponding to the anterior side, the third one to the minor curvature, the fifth to the posterior side, and the seventh to the major curvature of the aneurysmal wall. During the cardiac cycle, the left-ventricular motion determined a downward displacement of 8.2 mm for the ATAA (ascending aortic length of 10.5 cm). The ventricular traction resulted in a time-varying force with a maximum of 1.92 N at peak contraction and minimum of 1.28 N at heart relaxation. We also observed that stress magnitude in both circumferential and longitudinal directions decreased from the outer adventitial surface to the inner intimal surface in all quadrants. While the circumferential stress was uniformly distributed along the vessel periphery, the ATAA had maxima of longitudinal stress in the major curvature of the aneurysm wall. However, the right and posterior sides of the outer ATAA wall exhibited a transmural change of longitudinal stress with positive values at the inner surface to negative values on the outer surface.

**Figure 10:**
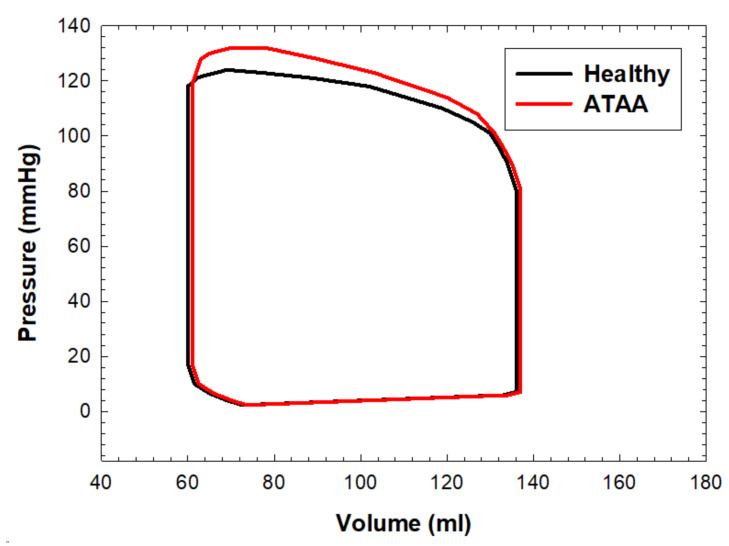
Pressure–volume loop of the left ventricle for the healthy and ATAA LHHMs.

The pressure–volume loop was calculated to quantify the left-ventricular function of the patient with ATAA (Figure 10). The relaxation, contraction, and passive filling were similar between models as no cardiac dysfunction was simulated for the left ventricle. The main difference was the slightly elevated systolic LV pressures for the ATAA LHHM systolic contraction as compared to that of the healthy aorta. As a measure of the vascular load, the valvulo-arterial impedance index was quantified as the ratio of the left-ventricular systolic pressure during ejection on the stroke volume indexed by body surface area. An 18% increase in the valvulo-arterial impedance index was observed for the ATAA LHHM with respect to that of the healthy aorta (valvulo-arterial impedance = 4.21 mmHg/mL/m^2^ for the healthy aorta, 4.95 mmHg/mL/m^2^ for the ATAA).

## 4. Discussion

In this study, we presented a computational framework for implementing the aneurysmal aorta in the LHHM as a first step for the development of a digital twin of a patient with ATAA. The framework included the patient-specific geometric segmentation and material parameter assessment of the ATAA wall, as well as the calibration of the global systemic circulation implemented in the LHHM. This led to a computational platform that can be used to reveal not only the biomechanics of the aneurysmal aorta but also its interplay with heart function. We demonstrated the key effect of ATAA motion on the resulting stress distribution at a level of detail hitherto impossible with other computational methods. Our findings demonstrate that the circumferential stress is uniformly distributed around the vessel periphery and that the longitudinal stretch is positive and highest on the vessel major curvature. The heart motion can lead to changes in the stress magnitudes through the aortic wall thickness, with compressive values in specific aortic quadrants of the outer ATAA surface. The proposed LHHM modeling and findings can take the in silico approach a step forward toward more realistic and accurate methods that will ultimately find their implementation for the clinical management of patients with ATAAs. 

The force driving the ATAA motion is the ventricular traction occurring every heartbeat, inducing the deformation and mechanical stress exerted on the vessel wall. Diagnostic imaging studies have demonstrated the downward displacement of the aortic root at systole and its return to previous position at diastole [40]. There are, however, few simulation studies of the interplay of the left ventricle with an ATAA because of technical challenges in cardiac modeling. Using an ideal aortic model, Beller et al. [28] simulated the aortic root motion by imposing an MRI-derived stretch and twist as boundary conditions. In the ascending aorta, the longitudinal stress was significantly increased as compared to an aortic model with uniform blood pressure as the sole boundary condition. Most importantly, the high longitudinal stress component was used to explain the clinical manifestation of aortic dissections where tearing occurs in the transverse direction because of increased wall stress level. Plonek et al. [41] also suggested that the risk is further exacerbated in the setting of a stiff aortic wall or hypertension in simplified aortic geometries under tensile loading. These findings on the key role of longitudinal stress on ATAA wall were corroborated in other studies [19,42] using patient-specific geometries but not considering the role of heart motion. Simulations adequately predicted the regions at high risk of tearing in patients who subsequently had surgical repair of dissected aortas. Our group demonstrated that peak longitudinal stress is significantly different between small and large ATAAs and between patients with either bicuspid or tricuspid aortic valve [25,43].

Emerging evidence suggests that ascending aortic length and elongation in ATAAs must be considered as a prognostic morphometric factor together with aortic size and body surface index to drive the timing for intervention in aneurysmal aortas. In a large patient cohort, Wu et al. [44] observed that an ascending aortic length of 11 cm serves as a potential intervention criterion for ATAA and is even more reliable than diameter because ascending aortic length is relatively immune to dissection. Aortic stretch may indicate the thinning of the aortic wall, fragmentation of elastin fibers, increase in the aortic pulse wave velocity indicating pronounced arterial stiffness, and asymmetrical flow profile portending abnormal wall shear stresses. In this study, we found that the heart kinematics determined a downward displacement of 8.2 mm at peak of heart contraction for the investigated patient with ascending aortic length of 10.5 cm. Heart contraction may determine a change in the through-the-thickness longitudinal stress component (from compressive to tensile) in specific quadrants of the ATAA wall. While the heart beating induces a stretch and twist of the aortic root, the supra-aortic vessels are constrained by the distal vasculature, likely portending a reaction force in the opposite direction of the aortic root downward displacement due to systolic contraction. The two forces—that due to the heart motion and that due to vasculature—are not aligned. Thus, the mid-ascending ATAA wall may undergo a bending loading as an arc-shaped specimen under tensile condition where the inner vessel surface has positive stress, and the outer surface has negative stress. This wall stress difference combined with the heterogeneous and anisotropic nature of the aorta can engender sufficient loading conditions to overcome the adhesive forces holding the aortic layers together and ultimately impart the onset of aortic dissection. This hypothesis needs further investigation, but the need for advanced numerical simulations is evident for a better understanding of the likelihood of aortic dissection. 

The heterogeneity of ATAAs includes different patterns of aortic dilatations and valvular morphologies, making surgeon predictions particularly challenging. Aortic diameter is now considered to have limited value for precise risk stratification because of the complex nature and clinical history of dilated aorta as governed by hemodynamic alterations and genetic pathways [44]. Providing therapies that are tailored to each patient is the broad goal for improving the clinical decision making in ATAAs. Recent studies proposed flow stress analysis [9], biomarker assays [18], and a combination of both [45] to overcome limitations of the aortic size paradox. In this context, the development of a digital twin could be a comprehensive virtual tool that uses mechanistic models to integrate the clinical data collected at in-hospital admission and over time for an individual. This may enable a new technology to improve patient management and efficacy of clinical trials for device design. The LHHM appears promising in this context since a personalized model can be generated starting from a highly realistic and accurate multiphysics model initially based on population-based geometric, biological, and biomechanical information about the human heart and great vessels. Several studies demonstrated the capability of LHHM to understand drug-related alterations in cardiac electrophysiology [46], the impact of the implantable cardioverter defibrillator [47] and left-ventricular assist device [48] on cardiac mechanics, the performance of the edge-to-edge repair of mitral valve [49], and transcatheter heart valve therapies in stenotic aortic valves [50]. Recently, Morany et al. [51] adapted the LHHM with a patient-specific aortic valve using an approach similar to that used in the present study. They demonstrated the role of kinematic heart behavior on the leaflet coaptation and stress distribution using an electromechanical model with more realistic boundary conditions. In a previous study, we adapted the left ventricle of LHHM using patient-specific CT scans and clinical data to calibrate the material constitutive behavior [39] and then quantify the intraventricular pressure and volume response under different degrees of aortic stenosis. The methodology here proposed to modify the aortic root and calibrate the left heart mechanics can be here integrated to simulate the ATAA in the setting of patients with bicuspid aortic valve or the dilated aortic root phenotype. Although we did not model the progress of aortic dilatation over time, the proposed LHHM could be easily adapted to account for growth and remodeling of the aortic wall over time.

### Study Limitations

The proposed ATAA LHHM has several limitations. The material parameters of ATAA wall were derived from ex vivo biaxial testing of collected human tissue, thereby limiting the application of LHHM to patients who underwent surgical repair. Additionally, the heterogeneous material properties of aortic layers were not simulated. A step toward clinical implementation of LHHM should be the calibration of population-based material parameters from preoperative CT images using inverse approaches [52]. Further studies should be done to confirm the transmural stress changes in different ATAAs, including the aortic shape patterns and valve phenotypes. Using structured mesh and high mesh refinement through the wall, the different material properties of both the medial and the adventitial layer could be integrated using data suggested by Sassani et al. [53]. A lumped-parameter model was used to account for the global flow and pressure of systemic circulation. However, cardiac MRI and computational flow analyses have clearly demonstrated the complex 3D nature of ATAA-related hemodynamic, leading to weakening and vessel remodeling [9,21]. Using the moving boundary method, the coupling of deforming heart geometries of LHHM with flow dynamics is feasible, as demonstrated by application in transcatheter aortic valve implantation [50]. The zero-stress configuration was not taken into account for the ATAA model, and this may impact the resulting stress distribution. However, the investigated patient was elderly; hence, aging likely decreased the influence of tethering force and residual stress on the aneurysmal biomechanical behavior. Moreover, the healthy LHHM model did not consider the potential biomechanical changes induced by aging on the material properties and fiber orientation. This likely changed the resulting stress distribution of the healthy aortic wall. The estimated ATAA displacements and boundary conditions were not validated against in vivo motion data extrapolated from MRI. Although the simulation of the heart mechanics leads to additional numerical challenges, this modeling approach allows revealing important insights into the major role of ventricular–arterial coupling in the pathophysiology of aortopathy. We envision a novel clinical strategy using computational stress analysis to improve the risk stratification of ATAAs. Physicians will reap the benefits of a novel paradigm of predictive medicine in which imaging and demographic data are used to tune the LHHM, and then reliable simulations allow rapidly quantifying human physiology and the risk associated with the ATAA.

## 5. Conclusions

Using the multiphysics modeling capability of LHHM, we analyzed the biomechanics of the aneurysmal ascending aorta and its interplay with cardiac movement. We conclude that, for ATAA biomechanics to be used for clinical risk stratification, more advanced numerical techniques, including for heart motion, are needed to understand longitudinal stress on ATAA. Models such as this will yield important data on ATAA biomechanics that can be implemented into clinical practice.
